# Poly-β-hydroxybutyrate administration during early life: effects on performance, immunity and microbial community of European sea bass yolk-sac larvae

**DOI:** 10.1038/s41598-017-14785-z

**Published:** 2017-11-08

**Authors:** Andrea Franke, Olivia Roth, Peter De Schryver, Till Bayer, Linsey Garcia-Gonzalez, Sven Künzel, Peter Bossier, Joanna J. Miest, Catriona Clemmesen

**Affiliations:** 1 GEOMAR Helmholtz Centre for Ocean Research Kiel, Evolutionary Ecology of Marine Fishes, Kiel, Germany; 2 Laboratory of Aquaculture and Artemia Reference Center, Ghent University, Ghent, Belgium; 3 Flemish Institute for Technological Research (VITO), Mol, Belgium; 4 Max Planck Institute for Evolutionary Biology, Department for Evolutionary Genetics, Plön, Germany; 5 Present Address: INVE Technologies N.V., Hoogveld 93, Dendermonde, Belgium; 6 Present Address: University of Greenwich, Department of Life & Sports Sciences, Chatham Maritime, London, United Kingdom

## Abstract

The reliable production of marine fish larvae is one of the major bottlenecks in aquaculture due to high mortalities mainly caused by infectious diseases. To evaluate if the compound poly-β-hydroxybutyrate (PHB) might be a suitable immunoprophylactic measure in fish larviculture, its capacity to improve immunity and performance in European sea bass (*Dicentrarchus labrax*) yolk-sac larvae was explored. PHB was applied from mouth opening onwards to stimulate the developing larval immune system at the earliest possible point in time. Larval survival, growth, microbiota composition, gene expression profiles and disease resistance were assessed. PHB administration improved larval survival and, furthermore, altered the larva-associated microbiota composition. The bacterial challenge test using pathogenic *Vibrio anguillarum* revealed that the larval disease resistance was not influenced by PHB. The expression profiles of 26 genes involved e.g. in the immune response showed that PHB affected the expression of the antimicrobial peptides ferritin (*fer*) and dicentracin (*dic*), however, the response to PHB was inconsistent and weaker than previously demonstrated for sea bass post-larvae. Hence, the present study highlights the need for more research focusing on the immunostimulation of different early developmental stages for gaining a more comprehensive picture and advancing a sustainable production of high quality fry.

## Introduction

In marine aquaculture, the stable and cost-effective production of high quality fish larvae is still hampered due to low survival during larval rearing, mainly caused by infectious diseases^1,2^. Early life stages of fish are highly susceptible to pathogens, because their immunocompetence is still severely limited^3^. For many aquaculture-relevant fish species, it has been reported that maternally-transferred immune molecules become mostly depleted during the first couple of days after hatch^4–7^. In European sea bass larvae, for example, maternal IgM was not detectable anymore by day 5 post hatch^8^. Accordingly, fish larvae mainly rely on their innate immune response, while adaptive immunity still needs to be acquired^9,10^. As a consequence, vaccines are not applicable during larval stages, as their effect depends on immunological memory, a feature of adaptive immunity^11^. Moreover, the standard method to combat diseases with antibiotics has selected for antibiotic-resistant bacteria, making treatments less effective and additionally being a risk to the environment and the human health^12^. Hence, investigating new biocontrol agents for disease prevention is crucial to evolve a sustainable aquaculture industry with enhanced larval survival rates and, therefore, reduced economic losses^13^. An alternative method is the stimulation of the immune response in fish larvae to improve their health and, thus, prevent the outbreak of diseases^14,15^.

A potential biocontrol strategy is the application of poly-β-hydroxybutyrate (PHB), the polymer of the short-chain fatty acid (SCFA) β-hydroxybutyrate (β-HB)^16^. PHB is an energy storage compound accumulated by a wide range of bacterial genera such as *Alcaligenes* and *Bacillus* ^17,18^. In various aquatic organisms, including shrimps (*Penaeus monodon*)^19^, rainbow trout (*Oncorhynchus mykiss*)^20^ and European sea bass (*Dicentrarchus labrax*) juveniles^21^, PHB has been demonstrated to increase survival and growth. Furthermore, dietary PHB increased the gastrointestinal (GI) microbial species evenness, diversity and richness in European sea bass juveniles and Siberian sturgeon (*Acipenser baerii*) fingerlings, which is suggested to contribute to a decreased infection risk^22,23^. The compound can be intestinally degraded into SCFAs, thereby, lowering the pH in the host’s gut^21^. While beneficial bacteria such as lactic acid bacteria profit from a lower intestinal pH, it has been shown that the cell growth and multiplication of pathogenic bacteria like *Vibrio* spp. is suppressed by SCFAs^24,25^. A disease protecting effect of PHB has been demonstrated, for example, in *Artemia franciscana* ^26^, Chinese mitten crab (*Eriocheir sinensis*) larvae^27^ and gnotobiotic Nile tilapia (*Oreochromis niloticus*) larvae^28^. Since SCFAs are known to play a central role in mammalian immunity, it is hypothesized that β-HB is capable of modulating the immune response in fish^29^. However, in teleosts PHB has so far only been shown to enhance the expression of immune-related genes in sea bass post-larvae^30^ and serum immune parameters as well as antibody response in adult Mozambique tilapia (*Oreochromis mossambicus*)^31^.

In the current study, it was investigated if the application of PHB modulates the immune response and the microbial community in European sea bass yolk-sac larvae. The aim was to stimulate the developing larval immune system at the earliest possible point in time. Therefore, freeze-dried PHB-accumulated bacteria (*Alcaligenes eutrophus*) were administered to sea bass larvae from mouth opening onwards and the effect on larval survival, growth performance, microbiota and disease resistance was assessed. Furthermore, an extensive analysis on the expression of genes involved in immunity, metabolism, growth and stress was performed.

## Materials and Methods

### Larval rearing

European sea bass (*Dicentrarchus labrax*) larvae were purchased from a commercial hatchery (Ecloserie Marine de Gravelines, France) at 3 dph and randomly distributed over 15 green tanks (100 larvae L^−1^) at GEOMAR Kiel, Germany. Each tank was filled with 30 L Baltic Sea water (5 μm-filtered and UV-treated) with an artificially increased salinity of 32 g L^−1^, which was gradually decreased to 26 g L^−1^ until 14 dph and increased to 32 g L^−1^ again thereafter to improve the efficiency of swim bladder inflation^32^. The water flow was gradually increased from 0.05 to 0.1 L min^−1^ until the end of the experimental period at 22 dph. The water temperature was increased stepwise from 15 to 18 °C and oxygen was maintained above 89% saturation throughout the experiment. Larvae were kept in the dark until first feeding at 8 dph and thereafter under a natural photoperiod regime (16 L: 8D). Tank bottoms were siphoned daily to remove dead larvae, feces and debris.

### Experimental treatments and feeding

Two different PHB doses (low and high) were administered from 2 different points in time onwards (mouth opening and first feeding), respectively, resulting in the following treatments: (1) Low PHB dose administered from mouth opening onwards (LMO), (2) Low PHB dose from first feeding onwards (LFF), (3) High PHB dose from mouth opening onwards (HMO), (4) High PHB dose from first feeding onwards (HFF) and (5) No PHB administration (Control). Each treatment was triplicated.

Amorphous PHB was administered in the form of freeze-dried PHB-accumulated bacteria (*Alcaligenes eutrophus*) produced by VITO (Mol, Belgium) as described in Thai *et al*. (2014)^33^. Bacteria with 2.5% and 75% PHB on cell dry weight (lowest and highest PHB accumulation possible for *A. eutrophus*) were used to prepare PHB solutions that were added to the tank water directly and used to enrich rotifers, respectively. From mouth opening (MO) at 5 dph onwards, PHB at a low or high dose was added directly to the tank water of the treatments LMO and HMO, respectively. For this purpose, the freeze-dried PHB-accumulated bacteria were suspended in UV-treated sea water with a salinity of 32 g L^−1^ and the PHB solutions were added daily at 10:00 h at a concentration of 10^6^ bacteria mL^−1^ tank water until the end of the experiment (22 dph). The water flow was turned off from 10:00 h to 18:00 h. Furthermore, all PHB treatments (LMO, HMO, LFF and HFF) were fed with PHB-enriched rotifers (*Brachionus plicatilis*) from first feeding (FF) at 8 dph onwards over a period of 14 days. The control treatment was fed on rotifers that were not enriched with PHB. Rotifers were reared in sterile filtered Baltic Sea water, fed on resuspended *Nannochloropsis* spp. concentrate and enriched with essential fatty acids (S.presso, INVE, applied according to instructions). Sea bass larvae were fed 3 times a day at 10:00 h, 15:00 h and 20:00 h (feeding ratio = 2:1:1). The rotifer density was increased over the course of the experiment from 4 to 12 rotifers mL^−1^ (first meal) and 2 to 6 rotifers mL^−1^ (second and third meal, respectively). For the PHB treatments, rotifers were enriched in freshly prepared PHB solutions at a density of 1000 ind. mL^−1^ for 30 min under gentle aeration directly before feeding. The PHB solutions for the rotifer enrichment consisted of freeze-dried PHB-accumulated bacteria with a low and high PHB content, respectively, suspended in sea water (UV-treated, salinity 32 g L^−1^) at a concentration of 10^8^ bacteria mL^−1^.

### Growth performance and survival

At 22 dph, 10 larvae were randomly sampled from each tank, killed with an overdose of MS 222, transferred into Eppendorf vials with sea water and immediately frozen on dry ice. Samples were stored at −80 °C. For growth analysis the total length (mm) of thawed larvae was measured. Afterwards larvae were briefly rinsed in distilled water to avoid salt residues, freeze-dried for 18 h at −55 °C and weighed in order to determine the larval dry weight (µg).

Furthermore, Fulton’s condition factor (K) was calculated according to equation (1):

$$K=\frac{W}{{L}^{3}}$$

where W equals the dry weight (µg) and L the total length (mm) of the larvae. For calculating survival rates, dead larvae were removed daily from the tanks and counted.

### Gene expression analysis

At 11 dph 18 larvae and at 22 dph 6 larvae were randomly sampled from each tank, killed with an overdose of MS 222, transferred into RNAlater and kept at 4 °C for 24 h before being stored at −20 °C. These 2 sampling points were chosen to assess short-term and mid-term effects of PHB administration on sea bass yolk-sac larvae. For the quantification of mRNA as a measure of gene expression levels, RNA of whole larvae was extracted using the RNeasy 96 Universal Tissue Kit (Qiagen) according to the manufacturer’s instructions. At 11 dph single larvae were too small to obtain enough RNA for gene expression measurements. Therefore, 6 × 3 larvae were pooled per tank for RNA extraction. At 22 dph 6 single larvae per tank were used. The RNA concentration was measured by spectrophotometry and normalized to a common concentration with RNase free water. RNA (500 ng) was reverse transcribed into cDNA, including a gDNA wipeout step (QuantiTect Reverse Transcription Kit, Qiagen). The cDNA was stored at −80 °C until further use. Primers for all genes were designed with Primer3 (version 0.4.0) using *D. labrax* sequences from GenBank or taken from the literature^34,35^ (Table 1). A qPCR BioMark™ HD System (Fluidigm) running a 96.96 Dynamic Array™ IFC (Gene Expression chip) was used to measure the expression profiles of 26 target genes as well as 3 reference genes in the larval samples. The chip run was performed using the GE Fast 96 × 96 PCR + Melt v2 thermal cycling protocol with a Tm of 60 °C according to the manufacturer´s instructions. For primer sequences, details on primer testing and the preparation of the Gene Expression chip see Franke *et al*. (2017)^30^.

**Table 1:** Name, abbreviation and function of the 26 genes of interest and 3 reference genes. Genes were divided into the following functional groups: (I) immunity, (II) growth and metabolism, (III) stress. Primers were either designed using sequences from GenBank (see accession number) or taken from literature (see reference).

Group		Abbreviation	Gene name and function	Acc. No./Ref.
**Immunity**	Innate immunity	*apoA1*	Apolipoprotein A1, antimicrobial protein	35
		*cc1*	CC chemokine 1, chemotactic cytokine	AM490065.1
		*cox2*	Cyclo-Oxygenase-2, pro-inflammatory enzyme	AJ630649.1
		*ifna1*	Interferon, cytokine	AM765846.2
		*il1b*	Interleukin 1 beta, pro-inflammatory cytokine	AJ311925.1
		*il8*	Interleukin 8, pro-inflammatory cytokine	AM490063.1
		*dic*	Dicentracin, antimicrobial peptide	AY303949.1
		*fer*	Ferritin, antimicrobial peptide	35
		*tlr1*	Toll-like receptor 1, pattern recognition receptor	KX399287
		*tlr9*	Toll-like receptor 9, pattern recognition receptor	KX399289
		*tnfa*	Tumor necrosis factor *α*, pro-inflammatory cytokine	DQ070246.1
	Adaptive immunity	*mhc class Ia*	Major Histocompatibility Complex I α, cell surface molecules	JX171695.1
		*mhc class IIa*	Major Histocompatibility Complex II α, cell surface molecules	FN667955.1
		*mhc class IIb*	Major Histocompatibility Complex II ß, cell surface molecules	AM113471.1
		*rag1*	Recombination activating protein 1, involved in VDJ recombination	FN687463.1
	Complement system	*c3*	Complement Component C3, classical & alternative pathway	HM563078.1
		*cla*	C-Lectin-A, lectin pathway	EU660935.1
		*gal*	Galectin, lectin pathway	EU660937.1
	Apoptosis	*casp3*	Caspase 3, protease	DQ345773.1
		*casp9*	Caspase 9, protease	DQ345776.1
**Growth & metabolism**		*gh*	Growth hormone	GQ918491.1
		*igf1*	Insulin-like growth factor 1	AY800248.1
		*fad6*	Fatty acid desaturase-6, fatty acid synthesis	FP671139.1
		*tryp*	Trypsin, protease	AJ006882.1
**Stress**		*cat*	Catalase, antioxidant	FJ860003.1
		*hsp70*	Heat shock protein 70, stress protection	AY423555.2
**Reference**		*actb*	Beta-actin	AJ537421.1
		*l13a*	Ribosomal protein L13 a	34
		*hsp90*	Heat shock protein 90	AY395632.1

### Larva-associated microbiota analysis

At 22 dph, 5 larvae were randomly sampled from each tank, killed with an overdose of MS 222, transferred into ethanol and stored at 4 °C. Since the larvae were too small to remove the intestinal tract, whole single larvae were used for DNA extraction (DNeasy 96 blood & tissue kit, Qiagen). Extracted DNA was stored at −20 °C until further use. For the 16S rRNA gene-based characterization of the larva-associated microbiota, DNA including a negative and a positive (*Vibrio* sp.) control was amplified (Phusion High-Fidelity DNA Polymerase, Thermo Fisher Scientific) using the primers F515 and R806 for the variable region 4 of the 16S rRNA gene^36^. Both primers contained adapters, barcodes, pad and linker sequences as described by Kozich *et al*. (2013)^37^. PCR cycling conditions for DNA amplification were as follows: 98 °C for 30 sec, followed by 30 cycles of 98 °C for 9 s, 55 °C for 15 sec and 72 °C for 20 sec, followed by 10 min at 72 °C. To eliminate primer dimers, the PCR products were purified using a MinElute 96 UF PCR purification kit (Qiagen). Subsequently, the DNA concentration of every sample was measured by spectrophotometry. Approx. 30 ng DNA per sample were pooled and a gel extraction was conducted (NucleoSpin gel and PCR clean-up kit, Macherey-Nagel). The extraction products were fluorometrically quantified (Qubit fluorometer, Invitrogen) and then pooled in equimolar amounts. Thereafter, the purified 16S rRNA amplicons were sequenced on a MiSeq sequencer (Illumina) as described in Kozich *et al*. (2013)^37^. MiSeq sequence data were assembled and filtered using mothur (version 1.16.1)^37^. Sequence reads were merged and aligned against the SILVA alignment database (release 119), all sequences that did not cover the variable region 4 were removed (SILVA alignment position 1968 to 11550)^38^. To reduce sequencing noise, a preclustering step (2 bp difference) was performed^39^ and chimeric sequences were removed using UCHIME as implemented in mothur^40^. The taxonomy of all sequences was estimated using the classify.seqs function in mothur against the RDP database^41^ using a bootstrap cutoff of 80%. The sequences were clustered at the 0.03 difference level to obtain operational taxonomic units (OTUs). Data have been submitted to the NCBI Sequence Read Archive under BioProject ID PRJNA384697. Furthermore, species richness, Simpson’s evenness and inverse Simpson’s diversity were calculated in mothur based on a dataset subsampled to a number of 10,000 reads per sample.

### Bacterial challenge test

In order to investigate the protective effect of PHB against vibriosis, sea bass larvae were exposed to pathogenic *Vibrio anguillarum* in a bath challenge. Therefore, 100 larvae were taken out of each tank (all treatments) at 22 dph and randomly distributed over 2 aquaria with a volume of 1.5 L (resulting in 50 larvae per aquaria). One aquarium was used for the bacterial challenge test (group BC) and the other one as an unchallenged control group (group UC). The experimental conditions were as follows: temperature 18 °C, salinity 32 g L^−1^, sea water was 5µm-filtered and UV-treated, photoperiod 16 L: 8D. The virulent *V. anguillarum* strain 87-9-117 was obtained from the Laboratory of Aquaculture and Artemia Reference Center (Ghent, Belgium) and preserved in 25% glycerol at −80 °C. Bacteria were cultured on 101 nutrient agar overnight at 25 °C. Subsequently, single colonies were picked and grown in 101 nutrient broth overnight at 25 °C and 200 rpm in an incubator shaker. Bacteria were harvested by centrifugation at 644 g for 10 min, washed twice in filtered and autoclaved sea water with a salinity of 32 g L^−1^ and added to the rearing water of group BC at a final density of 10^7^ CFU mL^−1^. After 24 h of challenge, the tank water was completely replaced in all aquaria. Afterwards the water flow was turned on overnight at a flow rate of 0.01 L min^−1^. The survival of the sea bass larvae was monitored over a period of 4 days. Larvae were not fed over the course of the experiment and dead larvae were removed daily.

### Enrichment of rotifers with 13 C labeled PHB-accumulated bacteria

To confirm bioencapsulation of the PHB-accumulated bacteria into the rotifers, bacteria had been labeled with 13 C during the accumulation of PHB into their biomass. To achieve 13 C labeling of the bacteria, they were produced by VITO (Mol, Belgium) as described in Thai *et al*. (2014)^33^ with the modification that 13 C labeled D-glucose (U-13CS, 99%, Eurisotop, France) was used as a carbon source for PHB accumulation. Subsequently, rotifers were enriched with the 13C labeled PHB-accumulated bacteria suspended in sea water (UV-treated, salinity 32 g L^−1^) at a density of 1000 rotifers mL^−1^ for 30 min under gentle aeration (rotifers without adding 13 C labeled PHB-accumulated bacteria were used as a control). After enrichment, rotifers were rinsed with Milli-Q water on a 50 µm sieve, transferred into tin foil cups and dried at 60 °C in a drying cabinet. Carbon isotope composition analysis of the samples was performed by isotope ratio mass spectrometry.

### Use of experimental animals

All experiments were approved by the ethical committee of Kiel University (Germany) under the file number V 312-7224.121-19 (24-2/13) and performed in accordance with the relevant guidelines and regulations.

### Statistical analyses

All statistical analyses were carried out in RStudio (version 0.98.1103). Survival data from the main experiment and the bacterial challenge test are presented by means of Kaplan-Meier curves and compared between treatment groups using a log-rank test (survival package)^42^. Post-hoc pairwise comparisons were performed in order to determine statistical differences between the respective treatments.

To analyze growth data, a mixed effect model, which included treatment as a fixed factor and tank as a random factor, was used (lme4 package)^43^. Growth data were tested for normality (Shapiro-Wilk test) and homogeneity of variances (Levene’s test). If the test assumptions were violated, data were Box-Cox transformed.

For gene expression analysis, the technical triplicates were used to calculate the mean cycle threshold value (Ct), the standard deviation (SD), and the coefficient of variation (CV) per sample. Samples with a CV larger than 4% were excluded from the analysis, as in accordance with Bookout and Mangelsdorf^44^. The expression stability of genes was calculated using qbase^+^ (Biogazelle) and the geometric mean Ct of the 3 most stable genes (reference genes *actb*, *l13a*, *hsp90*; M <0.5) was used to normalize the target genes (calculation of ΔCt-values). Permutational multivariate analyses of variance (PERMANOVA) were performed for each functional gene group to test for overall differences between the treatments. PERMANOVAs using ΔCt-values are based on Pearson distance matrices and were run with 999 permutations (adonis and Dist function, vegan and amap package)^45,46^. Multivariate effects were assessed on data averaged within an experimental tank. Subsequently, a mixed-effect model, which included treatment as a fixed factor and tank as a random factor, was used to analyze each individual target gene. Data were tested for normality (Shapiro-Wilk test) and homogeneity of variances (Levene’s test). If the test assumptions were violated, data were Box-Cox transformed.

Post-hoc pairwise comparisons were performed using the lsmeans function (lsmeans package)^47^. For graphical representation of gene expression data in response to PHB, the 2^−ΔΔCt^ method^48^ was applied by calculating the ΔΔCt for each target gene in relation to the mean ΔCt of the respective target gene in the untreated control. For graphical representation of the expression of all 26 target genes over the larval development, the 2^−ΔCt^ method^49^ was applied by using expression data of the untreated control only.

The microbiota data could not be analyzed for the whole data set due to an insufficient number of reads for many larval samples. Only samples with >10,000 reads were included in the statistical analysis, lowering the sample size to 1 to 4 larvae per tank and making it difficult to account for a potential tank effect. Hence, tank was not implemented in the statistical analyses, instead only treatment was included as a fixed factor using each larva instead of each tank as a replicate. To test for differences in the microbial community compositions between the treatments, bacterial phyla and OTUs were analyzed performing PERMANOVAs based on Euclidean distance matrices (adonis and vegdist function, vegan package, 999 permutations)^45^. In addition, a principle component analysis (PCA) for graphical visualization was implemented based on differences in the bacterial phyla composition according to the PHB treatments (ade4 package)^50^. To analyze the species richness, Simpson’s evenness and inverse Simpson’s diversity, analyses of variance (ANOVA) were performed (stats package)^51^.

### Data availability

Microbiota data have been submitted to the NCBI Sequence Read Archive under BioProject ID PRJNA384697. All other datasets generated and analyzed during the study are available at https://doi.pangaea.de/10.1594/PANGAEA.876665.

## Results

### Survival and growth performance

**Figure 1:**
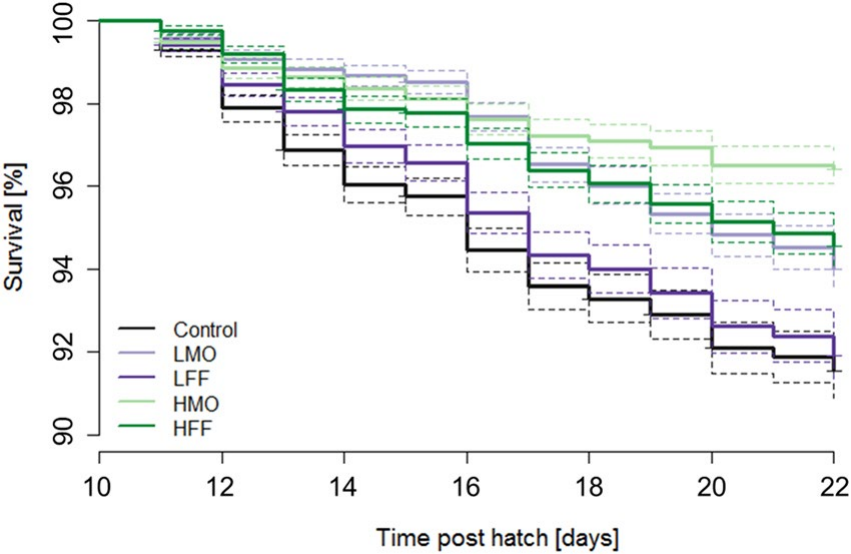
PHB administration enhances survival of sea bass larvae. Depicted are Kaplan-Meier survival curves of sea bass larvae (10 to 22 dph) administered a low PHB dose from mouth opening onwards (LMO), a low PHB dose from first feeding onwards (LFF), a high PHB dose from mouth opening onwards (HMO), a high PHB dose from first feeding onwards (HFF) or no PHB (control), respectively. The dashed lines represent the 95% confidence intervals.

Around first feeding (8 dph), the larval mortality was highly variable between the tanks across all treatments. Therefore, differences in survival were only examined after the onset of exogenous feeding. The treatments were significantly different from each other (χ^2^ = 175, df = 4, p < 0.001, Fig. 1). Larval survival in treatments LMO, HMO and HFF was significantly higher than in the control treatment (χ^2^_LMO_ = 37, χ^2^_HMO_ = 134, χ^2^_HFF_ = 56, df = 1, p < 0.001) while LFF and control (χ^2^_LFF_ = 1.1, df = 1, p = 0.30) were not significantly different from each other. Treatment HMO exhibited the highest survival rate.

The estimated growth-related parameters such as total length (mm), dry weight (µg) and Fulton’s condition factor K (µg mm^−3^) were not affected by the PHB treatment over the course of the experiment. The results are presented in Table 2.

**Table 2:** Growth-related parameters of sea bass larvae administered a low PHB dose from mouth opening onwards (LMO), a low PHB dose from first feeding onwards (LFF), a high PHB dose from mouth opening onwards (HMO), a high PHB dose from first feeding onwards (HFF) or no PHB (control), respectively. 10 larvae per tank were sampled at 22 dph. Values represent mean ± SEM. Furthermore, the mixed-effect model results are shown (F-value and p-value; degrees of freedom/residual degrees of freedom for all parameters: 4/10).

Growth-related parameters	22 dph (end of experiment)						
	Control	LMO	LFF	HMO	HFF	*F*	p
Dry weight (µg)	314 ± 18	386 ± 32	332 ± 25	378 ± 25	364 ± 23	0.7	0.61
Total length (mm)	8.0 ± 0.1	8.3 ± 0.2	8.2 ± 0.1	8.4 ± 0.2	8.4 ± 0.1	0.5	0.72
Condition^1^ (µg mm^−3^)	0.61 ± 0.03	0.63 ± 0.03	0.58 ± 0.03	0.62 ± 0.02	0.61 ± 0.03	0.6	0.66

^1^Fulton’s condition factor K = (W/L^3^).

### Gene expression

The expression of 26 genes involved in immune response, apoptosis, growth, metabolism, antioxidant activity and stress-response were analyzed and classified into the following functional gene groups: (I) immunity, (II) growth and metabolism, (III) stress. All genes included in the study (Table 1) were expressed at 11 and 22 dph (Suppl. Fig. S1).

The multivariate analysis showed that the expression of the immune-related genes differed significantly between the treatments at 11 dph (*F*_4,10_ = 5.0, p < 0.05) and 22 dph (*F*_4,10_ = 2.8, p < 0.05), while the other functional gene groups were not affected by the administration of PHB (Suppl. Table S1).

**Figure 2:**
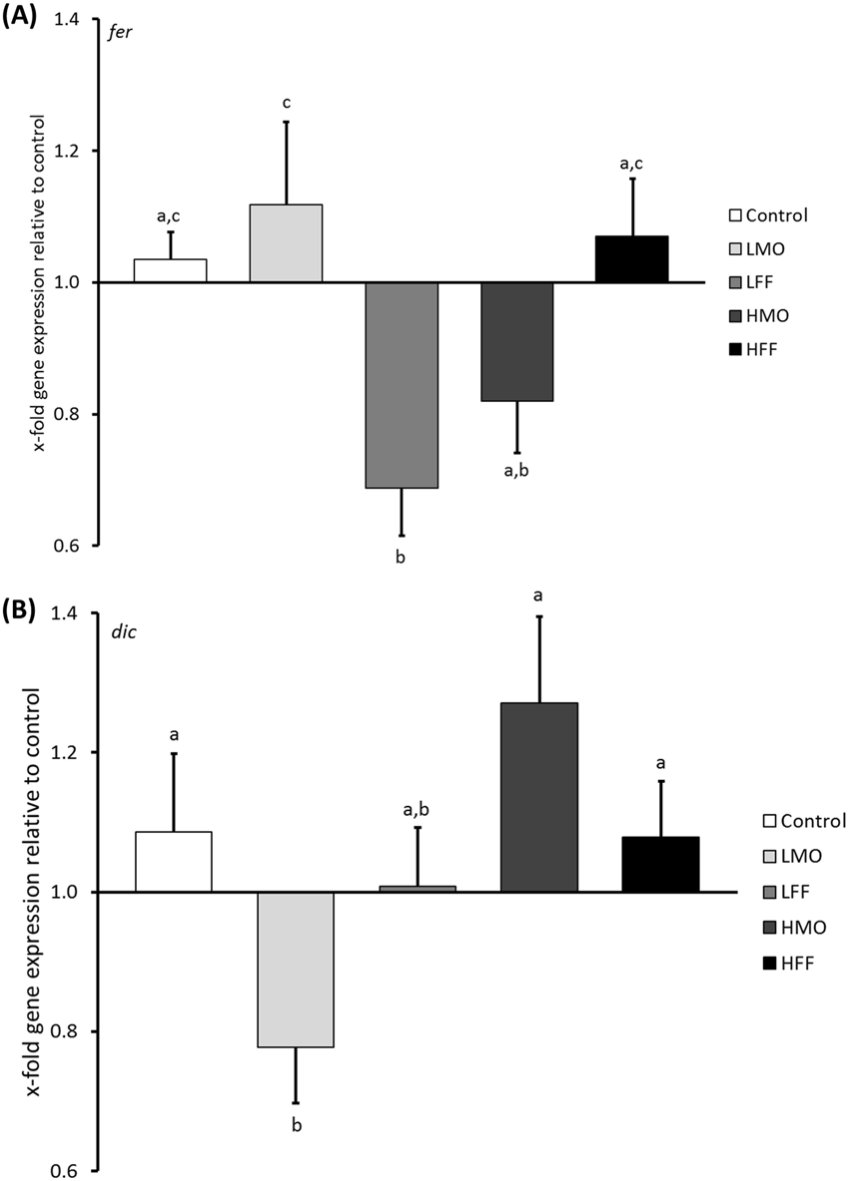
PHB affects the expression of (**A**) ferritin (*fer*) at 11 dph and (**B**) dicentracin (*dic*) at 22 dph in sea bass larvae. A low PHB dose from mouth opening onwards (LMO), a low PHB dose from first feeding onwards (LFF), a high PHB dose from mouth opening onwards (HMO), a high PHB dose from first feeding onwards (HFF) or no PHB (control) was administered, respectively. The figure displays the x-fold gene expression to the control. Data are presented as mean ± SEM. Treatments with different letters are significantly different at p < 0.05.

The subsequent univariate analyses (Suppl. Table S2) revealed that the impact of PHB on immune-related genes was driven by ferritin expression at 11 dph (*F*_4,10_ = 4.0, p < 0.05, Fig. 2A) and the dicentracin expression at 22 dph (*F*_4,10_ = 3.6, p < 0.05; Fig. 2B). The expression of ferritin was significantly down-regulated in treatment LFF (0.7 ± 0.07-fold, ΔCt = 2.6 ± 0.22) compared to the control (1.0 ± 0.04-fold, ΔCt = 1.8 ± 0.09). At this point in time (11 dph), the treatment LFF corresponded to 3 days of a low PHB dose encapsulated in the live feed. All other treatments were not statistically different from the control. The dicentracin expression was significantly decreased in treatment LMO (0.8 ± 0.08-fold, ΔCt = 3.0 ± 0.18) compared to the control (1.1 ± 0.11-fold, ΔCt = 2.5 ± 0.13). At this point in time (22 dph), the treatment LMO corresponded to 17 days of a low PHB dose administered via the tank water and 14 days of PHB encapsulated in the live feed. All other treatments were not statistically different from the control.

### Larva-associated microbiota

**Figure 3:**
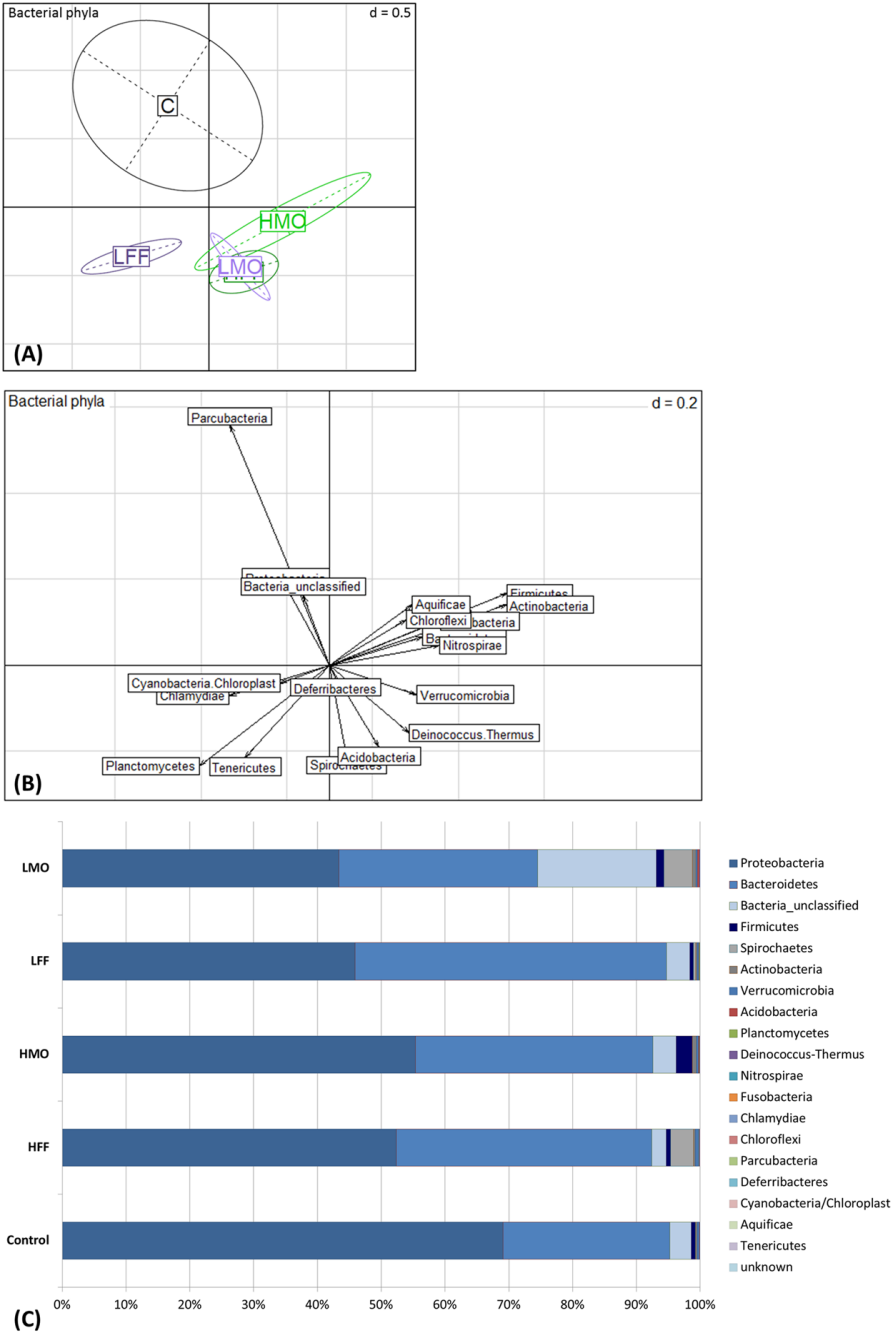
PHB controls the bacterial phyla composition in European sea bass larvae. (**A**) PCA plot visualizing the bacterial phyla composition in sea bass larvae (22 dph) administered a low PHB dose from mouth opening onwards (LMO), a low PHB dose from first feeding onwards (LFF), a high PHB dose from mouth opening onwards (HMO), a high PHB dose from first feeding onwards (HFF) or no PHB (control), respectively. (**B**) The corresponding scatterplot represents the contribution of each variable (bacterial phyla) to the total variability. Principle component 1 retains 15.5% and principle component 2 retains 12.9% of variance. (**C**) Percentage of different bacterial phyla in European sea bass larvae.

While the bacterial phyla composition differed significantly between the treatments (*F*_4,27_ = 1.9, p < 0.05, Fig. 3A and B), there was no difference at the OTU level (*F*_4,27_ = 1.0, p = 0.53). Bacteria belonging to the phyla Proteobacteria and Bacteroidetes accounted for more than 92.4% of the microbiota in all treatments except for LMO where they represented 74.5%. While unclassified bacteria accounted for 18.6% in the group LMO, they represented <3.7% in all other experimental groups. The phylum Firmicutes accounted for 2.5% and 1.3% of the bacterial community in the treatments HMO and LMO, respectively, while it was less than 0.8% in the all other groups (Fig. 3C). The species richness, Simpson’s evenness and inverse Simpson’s diversity were not affected by the PHB treatment (Table 3).

**Table 3:** Microbial community parameters of sea bass larvae administered a low PHB dose from mouth opening onwards (LMO), a low PHB dose from first feeding onwards (LFF), a high PHB dose from mouth opening onwards (HMO), a high PHB dose from first feeding onwards (HFF) or no PHB (control), respectively. Values represent mean ± SEM. Furthermore, ANOVA results are shown (F-value, p-value, degrees of freedom/residual degrees of freedom for all parameters: 4/27).

Microbial community parameters	Second sampling point (22 dph)						
	Control	LMO	LFF	HMO	HFF	*F*	p
Species richness	235 ± 25	268 ± 48	226 ± 13	272 ± 28	306 ± 8	0.3	0.86
Simpson’s evenness	0.04 ± 0.007	0.05 ± 0.009	0.04 ± 0.011	0.05 ± 0.014	0.03 ± 0.004	0.7	0.57
Inverse Simpson’s diversity	9 ± 1.8	12 ± 2.8	10 ± 2.4	16 ± 6.8	9 ± 1.4	0.7	0.59

### Bacterial challenge test

**Figure 4:**
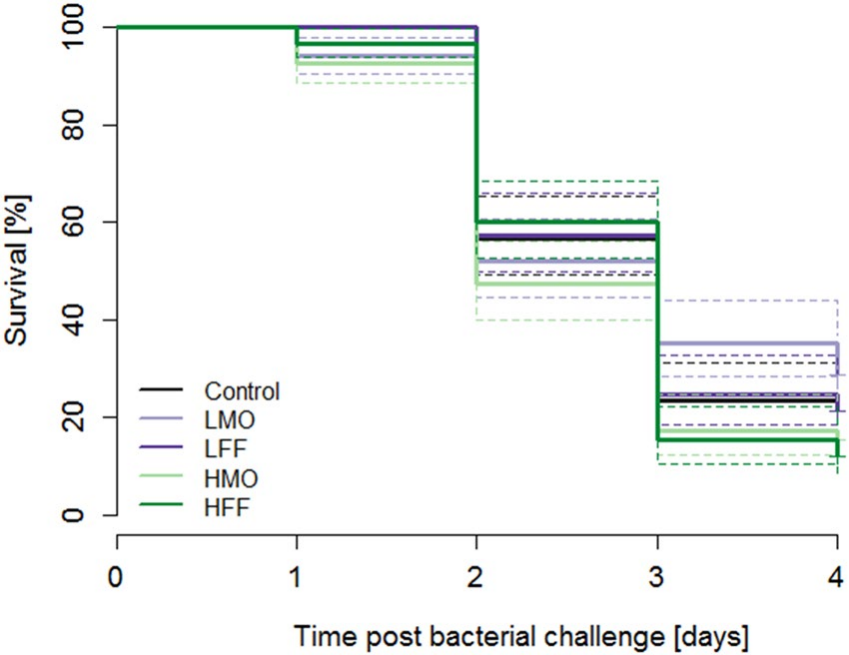
Larval survival differs between PHB treatments upon bacterial challenge test. Kaplan-Meier survival curves of sea bass larvae challenged with *V. anguillarum* at 22 dph after being administered a low PHB dose from mouth opening onwards (LMO), a low PHB dose from first feeding onwards (LFF), a high PHB dose from mouth opening onwards (HMO), a high PHB dose from first feeding onwards (HFF) or no PHB (control), respectively. The dashed lines represent the 95% confidence intervals.

Unchallenged sea bass larvae survived significantly better than larvae challenged with the *Vibrio anguillarum* strain 87-9-117 (χ^2^ = 900, df = 1, p < 0.001). The survival of the *V. anguillarum* challenged larvae differed from each other (χ^2^ = 10, df = 4, p < 0.05), however, there was no difference between the control group (no PHB) and the PHB treated groups (Suppl. Table S3). Solely, the survival of group LMO differed significantly from the groups HMO (χ^2^ = 6, df = 1, p ≤ 0.01) and HFF (χ^2^ = 5, df = 1, p < 0.05, Fig. 4), respectively.

### Enrichment of rotifers with 13 C labeled PHB-accumulated bacteria

The carbon isotope composition analysis revealed that rotifers supplemented with 13 C labeled PHB-accumulated bacteria had an average δ13 C value of 2635.6 ± 228.3‰ (SEM) confirming that they were highly enriched with PHB-accumulated bacteria. In contrast, rotifers not enriched with 13 C labeled PHB-accumulated bacteria had an average δ13 C value of −30.5 ± 0.1‰ (SEM).

## Discussion

In the present study, the effects of the bacterial energy storage compound PHB on sea bass yolk-sac larvae were examined with respect to survival, growth performance, immunity, microbial community and disease resistance. The larval stage is the most vulnerable of the life cycle stages^52,53^, suffering from highly variable mortality rates especially during critical developmental periods such as first feeding^54^. Fish larvae depend mainly on their innate immune response since their adaptive immune system is still developing, making them extremely susceptible to infections^55^. The maturation of the immune system is influenced by the intestinal microbiota, which starts to build up from mouth opening onwards and was shown to be altered by PHB supplementation^22,23,56,57^. Accordingly, the focus of this study was to investigate the impact of PHB on the developing larval immune system and microbiota, when administered at the earliest possible point in time (mouth opening).

In the present study, the survival was highly variable between the tanks independently of the respective treatment around the onset of first feeding. Similar results were obtained in another study with sea bass larvae where the highest mortality rates occurred between 6 and 10 dph around the initiation of exogenous feeding^58^. As this variability would have masked any treatment effect, survival was statistically assessed only at the end of the critical first feeding period. The survival was significantly higher in the treatments LMO, HMO and HFF compared to the control whereby the administration of PHB in a high dose from mouth opening onwards (HMO) resulted in the best larval survival. The larval growth performance was not significantly affected by PHB administration. However, there was a trend towards a higher larval weight in all PHB treatments compared to the control. Larvae that received either a low or high PHB dose from mouth opening onwards (LMO and HMO) tended to have highest weights at the end of the experiment. Treatments LMO and HMO were the only experimental groups being provided with PHB via the tank water before larvae were able to feed. These results indicate that the ingestion of PHB before the onset of first feeding through drinking has the potential to increase the performance of sea bass larvae (either by providing energy or indirectly by changing the intestinal microbiota). An improvement in survival and growth after PHB administration was also found in blue mussel (*Mytilus edulis*) larvae^59^, Chinese mitten crab (*Eriocheir sinensis*) larvae^60^, giant tiger prawn (*Penaeus monodon*) post-larvae^19^ and sea bass juveniles^21^. It is hypothesized that PHB is gastrointestinally degraded into the SCFA β-HB (by digestive enzymes, PHB degrading bacteria or both), which promotes growth and survival by being an additional carbon source for the host^16,61^. SCFAs are known to be the main energy source of intestinal cells mediating their proliferation, differentiation and mucin production^62^. Moreover, SCFAs lower the intestinal pH, which may enhance the activity of digestive enzymes leading to a better nutrient absorption^63^. This additional energy might be the reason for the observed enhanced survival in sea bass larvae provided with PHB.

The expression of 26 genes involved in the innate and adaptive immune response, the complement system, apoptosis, digestion, growth and oxidative damage was analyzed in order to estimate the effect of PHB on larval immunity, metabolism and stress. It has to be noted that PHB was administered in form of freeze-dried (non-viable) PHB-accumulated bacteria and that a direct effect of these bacteria on the larval immune system cannot be excluded. However, previous studies using bacteria accumulated with different PHB doses showed that the level of PHB was most likely an important driver for the observed effects (e.g. disease resistance)^19^.

In the current study, the larval immune gene expression profiles differed significantly between the PHB treatments at 11 and 22 dph, respectively. The analyses of individual genes revealed that this effect was only driven by a differential expression of the antimicrobial peptides ferritin (*fer*) and dicentracin (*dic*). This indicates that the observed changes in immune gene expressions are moderate and, therefore, cannot be detected for most individual genes. The differences in the expression of *fer* and *dic* between the treatments did not show a consistent pattern as observed in sea bass post-larvae, where several immune-related genes were up-regulated in response to PHB administration^30^. However, it has to be taken into account that the methodological approach only allowed to analyze the expression of a limited number of genes (26) at 2 time points. Since the immune response in fish early life stages is mediated by a complex network of innate defense mechanisms^3^, it cannot be excluded that PHB may have induced changes in the expression of immune genes that were not assessed in this study. It was hypothesized that the metabolic product of PHB, the SCFA β-HB, might stimulate the immune system in fish in a similar way as in mammals by binding to G protein-coupled receptors (GPRs)^29^. Mammalian GPR43 for example recognizes butyrate and is highly expressed in macrophages and neutrophils^64^. Both cell types are known to be present already in fish early life stages^65^. The immune response might be triggered in addition through changes in the intestinal microbial community caused by the degradation of PHB lowering the luminal pH^16^. A lowered GI pH might result in the inhibition of virulence factor production and growth of pathogenic bacteria and promote the multiplication of specific beneficial bacteria, which can activate the humoral and cellular immune system through the recognition of microbe-associated molecular patterns (MAMPs) by pattern-recognition receptors (PRRs)^57,66,67^. In Siberian sturgeon larvae administered PHB from first-feeding onwards, the intestinal microbiota was altered^68^, however, the larval immune response was not examined. In the current study, the larva-associated bacterial phyla composition differed between the treatments indicating that PHB has the potential to trigger an immune response by altering the MAMPs (as described above). Interestingly, the treatment HMO, exhibiting the best survival and a trend towards an increased growth performance, had the highest proportion of bacteria belonging to the phylum Firmicutes. The phylum includes a variety of probiotic bacteria such as *Bacillus* spp. that have been demonstrated to confer a health benefit to the host^15,57^. The larval bacterial communities on the level of operational taxonomic units (OTUs) as well as the bacterial species richness, evenness and diversity were not affected by the administration of PHB. In contrast, the application of PHB increased the bacterial evenness and diversity in sea bass juveniles^21,22^ and Siberian sturgeon juveniles^23^. These results indicate that the mode of action of PHB might vary between different life cycle stages and, thus, depend on the maturity of the GI microbiota. However, since the microbiota could only be analyzed for a small number of replicates (due to an insufficient number of MiSeq reads for many larval samples), further microbiota analyses should be performed in future studies.

In previous studies, it was demonstrated that PHB increased disease resistance in aquatic organisms. Survival was enhanced when PHB was provided during bacterial challenge tests with axenic Nile tilapia larvae^28^, Chinese mitten crab larvae^27^ and *Artemia* ^24,26^. It was concluded that PHB inhibited the multiplication of the pathogenic bacteria in the GI tract. SCFAs such as β-HB pass bacterial cell membranes and release protons in the cytoplasm, which have to be exported in order to maintain cellular homeostasis. This energy consuming process might suppress the growth of pathogenic bacteria^16^. In the current study, we investigated if the immunomodulating effects of PHB may increase the robustness of sea bass larvae during a *V. anguillarum* infection. In order to disentangle the immediate antimicrobial effect of the compound and its immunostimulatory capacities, sea bass larvae were not supplied with PHB during the bacterial challenge test. Compared to the control, none of the PHB treatments had a significant effect on the survival of sea bass larvae challenged with pathogenic bacteria which is the desired objective in commercial fish larviculture. In contrast, when shrimp (*Penaeus monodon*) post-larvae were infected with pathogenic bacteria after being fed with a PHB-supplemented diet, survival was increased compared to the control^19^. The divergent results obtained in sea bass larvae and shrimp post-larvae might be attributed to the different degrees of virulence of the bacteria used for the challenge tests. While over 75% of the *Vibrio* challenged sea bass larvae died in the control treatment (no PHB), less than 40% of the *Vibrio* challenged shrimp post-larvae died in the control treatment (no PHB). Therefore, the high virulence of the *V. anguillarum* strain used in the present study might have masked the potential protective effect of PHB. Furthermore, possible differences in the mode of action of PHB between teleost fish and crustaceans have to be taken into consideration and addressed in future studies.

In conclusion, our study indicates that administering amorphous PHB to sea bass larvae already before the onset of first feeding via the tank water had a positive influence on their survival. A prolongation of the time span of the PHB application is advised for further research to investigate if a significant growth-promoting effect can be obtained. The impact of PHB on the immune gene expression of sea bass yolk-sac larvae was inconsistent and weaker than detected in sea bass post-larvae^30^. During their first weeks of life, fish larvae undergo significant morphological and physiological changes including the maturation of the immune system. Thus, the mode of action of PHB in early, respectively, late larval stages might vary substantially. The current study highlights the need for more research focusing on the immunostimulation at different points in time during early development. In order to elucidate life-stage dependent differences, future studies on fish larvae analyzing an extended set of immune-related genes are necessary.

## Electronic supplementary material

Supplementary Dataset 1
