# Supplementary material for: Poly-β-hydroxybutyrate administration during early life: effects on performance, immunity and microbial community of European sea bass yolk-sac larvae
Source: Sci Rep. 2017 Nov 8;7:15022. doi: 10.1038/s41598-017-14785-z (PMC5678127; doi:10.1038/s41598-017-14785-z)
Supplement: Supplementary file 1 — Supplementary Dataset 1 [file 41598_2017_14785_MOESM1_ESM.doc]

**Poly-β-hydroxybutyrate administration during early life: effects on performance, immunity and microbial community of European sea bass yolk-sac larvae**

Andrea Franke, Olivia Roth, Peter De Schryver, Till Bayer, Linsey Garcia-Gonzalez, Sven Künzel, Peter Bossier, Joanna J. Miest and Catriona Clemmesen

**Supplementary material**


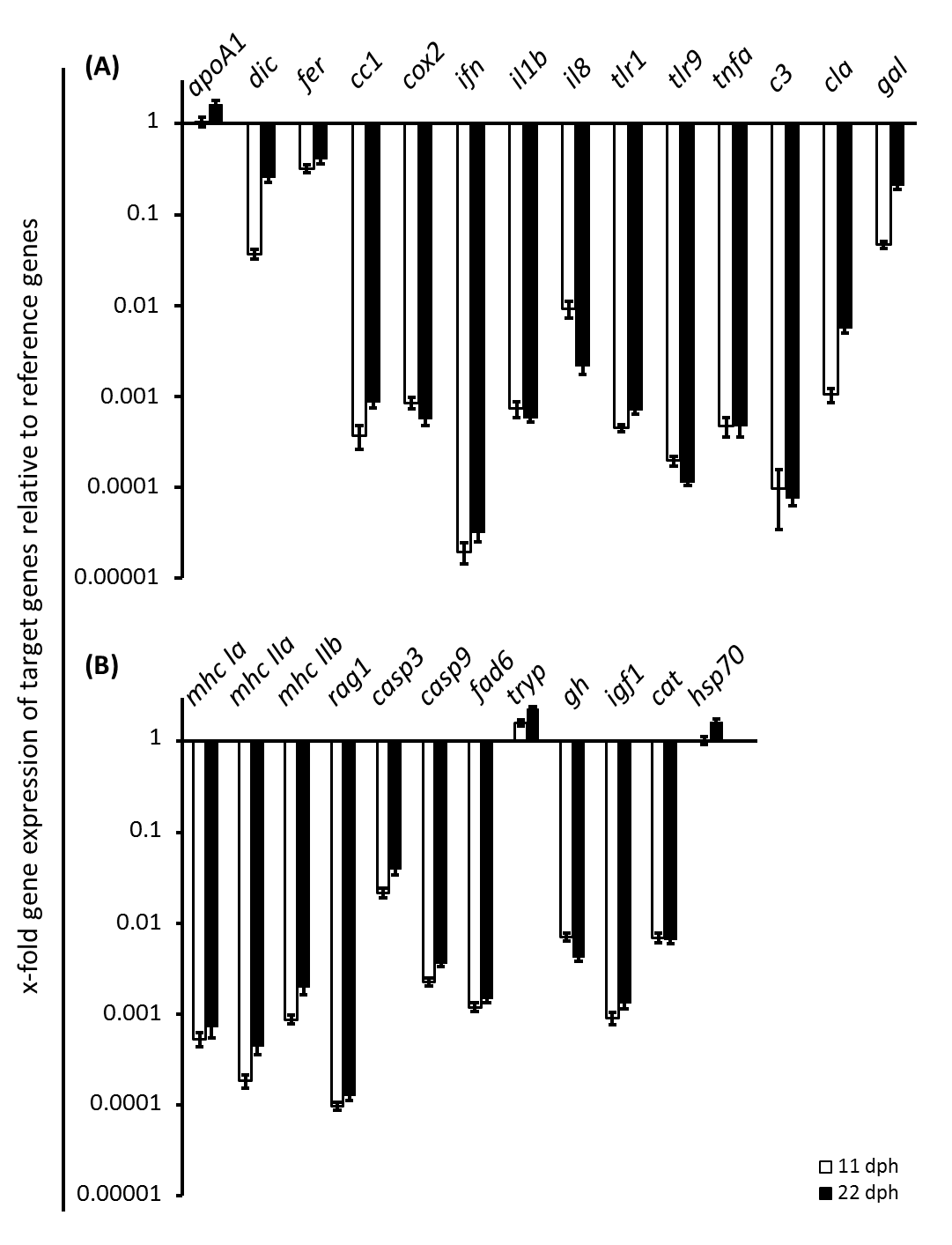


**Fig. S1.** Expression of all target genes in European sea bass larvae at 11 dph (white) and 22 dph (black) in the untreated control. **(A)** Genes involved in innate immunity and complement system, **(B)** adaptive immunity, apoptosis, metabolism, growth and stress. The figures display the x-fold gene expression of the target genes relative to the geometric mean Ct of the reference genes. Data are presented as mean ± SEM. For information on the gene functions see Table 3.

**Table S1**

PERMANOVA results for larval gene expression profiles (based on a Pearson distance matrix). The effect of the experimental treatment (PHB administration) on overall gene expression levels was tested for different functional gene groups. *F*-statistics and p-values are shown. Degrees of freedom/residual degrees of freedom: 4/10.

|  | 11 dph | |  | 22 dph | |
| --- | --- | --- | --- | --- | --- |
| **Functional group** | *F* | p |  | *F* | p |
| Immunity | 5.0 | **0.018** |  | 2.8 | **0.046** |
| Growth & metabolism | 1.4 | 0.39 |  | 1.1 | 0.49 |
| Stress | 0.6 | 0.59 |  | 0.8 | 0.67 |

**Table S2**

Univariate statistical results (mixed-effect model) for all genes of functional group (I) immunity. *F*-values and p-values are shown. Degrees of freedom/residual degrees of freedom: 4/10.

|  | 11 dph | |  | 22 dph | |
| --- | --- | --- | --- | --- | --- |
| **Gene** | *F* | p |  | *F* | p |
| *apoA1* | 3.04 | 0.07 |  | 1.31 | 0.33 |
| ***dic*** | 1.52 | 0.27 |  | 3.60 | **0.04** |
| ***fer*** | 4.02 | **0.03** |  | 1.66 | 0.23 |
| *cc1* | 2.78 | 0.09 |  | 1.63 | 0.24 |
| *cox2* | 1.03 | 0.44 |  | 0.78 | 0.56 |
| *ifna1* | 1.98 | 0.17 |  | 1.09 | 0.41 |
| *il1b* | 2.28 | 0.13 |  | 0.43 | 0.78 |
| *il8* | 3.04 | 0.07 |  | 2.07 | 0.16 |
| *tlr1* | 0.65 | 0.64 |  | 1.38 | 0.31 |
| *tlr9* | 1.55 | 0.26 |  | 0.83 | 0.54 |
| *tnfa* | 1.25 | 0.35 |  | 2.06 | 0.16 |
| *c3* | 0.24 | 0.91 |  | 0.24 | 0.91 |
| *cla* | 1.54 | 0.26 |  | 2.29 | 0.13 |
| *gal* | 0.18 | 0.95 |  | 2.41 | 0.12 |
| *mhc class Ia* | 0.37 | 0.83 |  | 1.48 | 0.28 |
| *mhc class IIa* | 0.35 | 0.84 |  | 1.12 | 0.40 |
| *mhc class IIb* | 0.87 | 0.51 |  | 2.00 | 0.17 |
| *rag1* | 0.91 | 0.49 |  | 0.08 | 0.99 |
| *casp3* | 0.62 | 0.66 |  | 1.70 | 0.23 |
| *casp9* | 0.33 | 0.85 |  | 1.29 | 0.34 |

**Table S3**

Survival analyses for larvae challenged with *Vibrio anguillarum* (bacterial challenge test). The different treatment groups were compared with each other performing log-rank tests (df = 1). *χ^2^*-values and p-values are presented.

| Treatments | *χ^2^* | p |
| --- | --- | --- |
| C - LMO | 0.89 | 0.34 |
| C - LFF | 0.04 | 0.84 |
| C - HMO | 2.93 | 0.09 |
| C - HFF | 1.61 | 0.21 |
| LMO-LFF | 0.59 | 0.44 |
| LMO-HMO | 6.47 | **0.01** |
| LMO-HFF | 5.42 | **0.02** |
| LFF - HMO | 3.74 | 0.06 |
| LFF - HFF | 2.24 | 0.13 |
| HMO - HFF | 0.46 | 0.50 |
